# Use of Aldosterone Antagonist to Treat Diarrhea and Hypokalemia of Ogilvie's Syndrome

**DOI:** 10.1155/2016/1207240

**Published:** 2016-10-12

**Authors:** Pradhum Ram, Abhinav Goyal, Marvin Lu, Joshua Sloan, William McElhaugh

**Affiliations:** Einstein Medical Center, Philadelphia, PA, USA

## Abstract

Ogilvie's syndrome (OS) is a functional obstruction of the bowel due to an autonomic imbalance. It often presents with diarrhea and is associated with hypokalemia. We present a case of a 70-year-old male who developed severe abdominal distension, watery diarrhea, and persistent hypokalemia status after left hip arthroplasty after suffering from a femoral neck fracture due to a fall and was diagnosed with OS. The persistent hypokalemia was slow to improve despite aggressive repletion because of the high potassium losses in the stool. This is most likely mediated through the increased expression of BK channels in the colonic mucosa. Aldosterone is theorized to have a role in the regulation of BK channels. Spironolactone was subsequently given and resulted in marked improvement of the diarrhea and hypokalemia. Thus, this case suggests a novel therapeutic approach for the treatment of Ogilvie's syndrome-associated diarrhea and hypokalemia.

## 1. Introduction

Ogilvie's syndrome is primarily a functional obstruction of the bowel. There is no mechanical intra- or extraluminal obstruction and the syndrome is thought to develop from an autonomic imbalance between the sympathetic and parasympathetic systems [1–3]. It has been associated with various electrolyte abnormalities including hypokalemia and metabolic acidosis. It is usually managed conservatively with physical therapy, electrolyte repletion, and encouraging ambulation to stimulate the bowels. Aldosterone antagonists have not been used previously to treat the hypokalemia arising from Ogilvie's syndrome. We present a case of Ogilvie's syndrome-associated diarrhea and hypokalemia that was successfully treated with spironolactone.

## 2. Case

**Figure 1:**
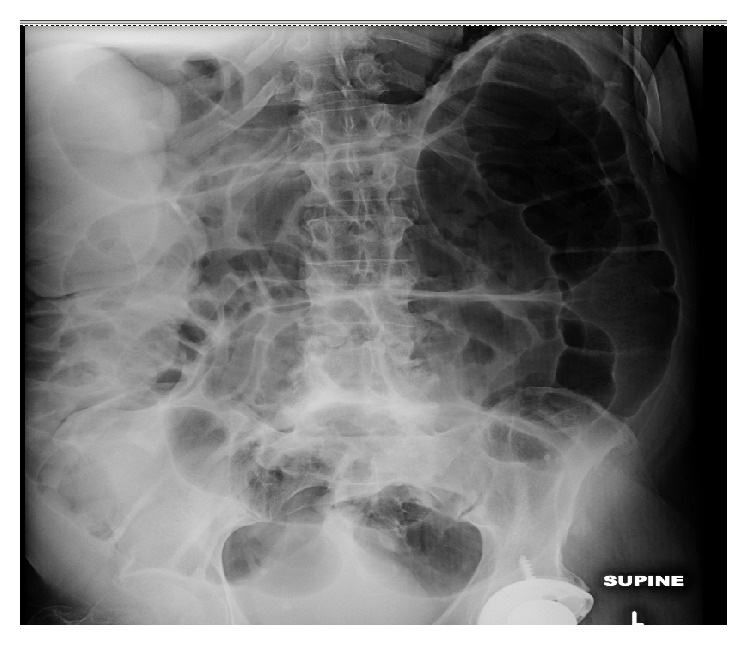
Abdominal X-ray showing severe colonic dilation due to pseudoobstruction.

**Figure 2:**
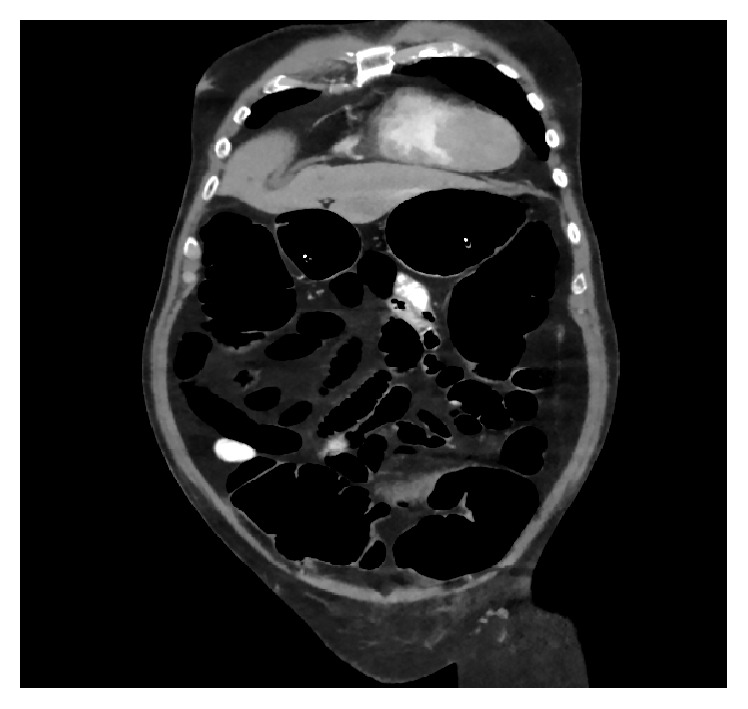
CT image of abdomen showing marked colonic dilation without any evidence of obstruction.

A 70-year-old African-American male with a history of hypertension, COPD, cryptogenic cerebrovascular accident, and paroxysmal atrial fibrillation presented after he was hit by a van whilst crossing a road. On admission, his physical exam was remarkable for mild abdominal distention and an externally rotated left lower extremity. He was diagnosed with a nondisplaced fracture of the left femoral neck, for which he underwent a total hip arthroplasty. Following the arthroplasty, his hospital course was complicated by a pulmonary embolus, which was treated with low molecular weight heparin and warfarin. Later on, during his hospital stay, he developed watery diarrhea and marked abdominal distension with hypoactive bowel sounds. He did not have any nausea, vomiting, or abdominal pain.* Clostridium difficile* colitis was ruled out by a PCR based assay. Abdominal X-ray and CT abdomen showed marked gaseous distension of large bowel (Figures 1 and 2). At this point, he was diagnosed with Ogilvie's syndrome. He subsequently underwent 2 successive decompressive sigmoidoscopies, which resulted in minimal improvement of his abdominal distention. His colon enlarged to a maximum diameter of 12 cm. Lab results were remarkable for hypokalemia to 2.8 mEq/L. Despite aggressive repletion with over 160 mEqs of potassium a day his serum potassium remained at <3 mEq/L after 4 days. Due to this persistent hypokalemia urine and stool electrolyte studies were obtained. His urinary potassium was <10 mEq/L, stool sodium was <15 mEq/L, and stool potassium was elevated to >140 mEq/L. This pattern was consistent with Ogilvie's syndrome. At this point, besides continued potassium repletion and ambulation as tolerated with the help of the physical therapist, he was also started on spironolactone 50 mg twice a day to help decrease stool losses. After starting spironolactone his potassium levels improved and his stool output decreased gradually over the next 3-4 days. His potassium remained stable and he was discharged to a skilled nursing facility for further physical therapy. On follow-up, 20 days after discharge his potassium was found to be stable at 5.1 and his colonic distention had improved.

## 3. Discussion

Ogilvie's syndrome when initially described by Sir William Ogilvie was thought of as a result of sympathetic deprivation arising from invasion of the colonic wall [2]. However, over the past few decades, it has been more commonly associated with a disturbance in parasympathetic supply [3]. Most cases present with diarrhea, although constipation has been described albeit rarely. It is frequently associated with electrolyte abnormalities like hypokalemia and metabolic acidosis. Our patient had severe persistent hypokalemia due to high stool potassium losses. This was confirmed by the low urinary concentration of potassium combined with high stool potassium concentration in our patient. Despite aggressive repletion it failed to improve given the continued losses in stool. Since, hypokalemia can worsen a preexisting ileus it is likely that it contributed to the slow bowel recovery in our patient as well.

Potassium is primarily an intracellular ion. A variety of channels, the sodium-potassium ATPase and Na/K/Cl cotransporter, are responsible for maintaining potassium homeostasis in our body. The role of other channels like maxiK or BK channels has been less clearly defined. They have been known to be overexpressed in the colon in end-stage renal disease patients where the body relies heavily on the gastrointestinal tract to eliminate potassium since the kidneys cannot adequately do so [3, 4]. However, they are now increasingly thought to be responsible for hypersecretion of potassium, which drives the osmotic diarrhea in colonic pseudoobstruction [3, 5]. In vitro studies have shown that these channels are upregulated by the cAMP pathway and thus may be affected by hormones likely aldosterone and somatostatin [3, 5]. Somatostatin was shown to decrease stool output in a similar patient with colonic pseudoobstruction in a previous case report [6].

Aldosterone is an important mediator of potassium regulation and it is well known that through its effects on the kidneys aldosterone increases urinary excretion of potassium. Due to this reason, aldosterone antagonists have long been used to treat hypokalemia associated with primary hyperaldosteronism. Aldosterone, however, has never been used to treat diarrhea and hypokalemia arising from diarrhea, especially in Ogilvie's syndrome. There is only one case report where spironolactone was used to treat cholera and it was shown to have significantly reduced stool sodium and potassium excretion [7]. We chose spironolactone over somatostatin due to significantly lower risk of adverse effects associated with it. Spironolactone not only improved serum potassium in our patient but also helped reduce the stool output, likely due to the fact that diarrhea in colonic pseudoobstruction is driven by hypersecretion of potassium.

In conclusion, this case presents a novel therapeutic option for the treatment of diarrhea and electrolyte abnormalities associated with Ogilvie's syndrome. Prospective studies will be needed in the future to explore the effect of aldosterone on diarrhea, stool potassium losses, and Ogilvie's syndrome.
